# Effects of an Early Experience of Reward through Maternal Contact or its Denial on Laterality of Protein Expression in the Developing Rat Hippocampus

**DOI:** 10.1371/journal.pone.0048337

**Published:** 2012-10-31

**Authors:** Androniki Raftogianni, Antonios Stamatakis, Angeliki Papadopoulou, Konstantinos Vougas, Athanasios K. Anagnostopoulos, Fotini Stylianopoulou, George Th. Tsangaris

**Affiliations:** 1 Laboratory of Biology-Biochemistry, Department of Basic Sciences, School of Health Sciences, University of Athens, Athens, Greece; 2 Biomedical Research Foundation of the Academy of Athens (BRFAA); Proteomics Research Unit, Athens, Greece; University of Medicine & Dentistry of NJ - New Jersey Medical School, United States of America

## Abstract

Laterality is a basic characteristic of the brain which is detectable early in life. Although early experiences affect laterality of the mature brain, there are no reports on their immediate neurochemical effects during neonatal life, which could provide evidence as to the mechanisms leading to the lateralized brain. In order to address this issue, we determined the differential protein expression profile of the left and right hippocampus of 13-day-old rat control (CTR) pups, as well as following exposure to an early experience involving either receipt (RER) or denial (DER) of the expected reward of maternal contact. Proteomic analysis was performed by 2-dimensional polyacrylamide gel electrophoresis (PAGE) followed by mass spectroscopy. The majority of proteins found to be differentially expressed either between the three experimental groups (DER, RER, CTR) or between the left and right hemisphere were cytoskeletal (34%), enzymes of energy metabolism (32%), and heat shock proteins (17%). In all three groups more proteins were up-regulated in the left compared to the right hippocampus. Tubulins were found to be most often up-regulated, always in the left hippocampus. The differential expression of β-tubulin, β-actin, dihydropyrimidinase like protein 1, glial fibrillary acidic protein (GFAP) and Heat Shock protein 70 revealed by the proteomic analysis was in general confirmed by Western blots. Exposure to the early experience affected brain asymmetry: In the RER pups the ratio of proteins up-regulated in the left hippocampus to those in the right was 1.8, while the respective ratio was 3.6 in the CTR and 3.4 in the DER. Our results could contribute to the elucidation of the cellular mechanisms mediating the effects of early experiences on the vulnerability for psychopathology, since proteins shown in our study to be differentially expressed (e.g. tubulins, dihydropyrimidinase like proteins, 14-3-3 protein, GFAP, ATP synthase, α-internexin) have also been identified in proteomic analyses of post-mortem brains from psychiatric patients.

## Introduction

Early experiences have a determinant role on brain development and thus affect brain function and expression of behavior, as well as vulnerability for psychopathology, throughout the lifespan. Although this has been clearly documented in both humans and animal models through a large number of studies [1], [2], [3], [4], many questions as to the underlying mechanisms mediating the effects of early experiences on the developing brain still remain unanswered. In an effort to address such a question in the present study we investigated the effect of exposure to an early experience involving receipt or denial of the expected reward of maternal contact, a model developed in our laboratory [5], [6], on the proteome of the hippocampus, of 13-day-old rat pups. More specifically, rat pups on postnatal days 10–13 are exposed to a T-maze, one arm of which leads to the mother-containing cage to which pups of the one group (Receiving Expected Reward-RER) are permitted access, upon reaching its entrance. In contrast, pups of the other group are denied entry and thus the expected reward of contact with the mother (Denied Expected Reward-DER). These two experiences have been shown to affect the pups at the cellular level of the brain, as well as the behavioral, both immediately after the experience on postnatal day 13 5,6 and in adulthood [6], [7]. In particular the DER experience has been shown to lead to increased activation of the hippocampus of 13-day-old pups [5], as well as in adulthood, following the Morris Water Maze, a hippocampus-dependent behavioral task, in which the DER animals exhibited better mnemonic abilities [6]. Based on these results in the present study we focused on the analysis of the hippocampal proteome of 13-day-old DER, RER, and control (not exposed to any early experience) rat pups, in an effort to identify proteins differentially expressed as a result of the early experience. Indeed previous studies have shown that maternal separation in rats [8], [9] alters the adult hippocampal proteome. However it must be pointed out that in contrast to this early experience, which is highly distressful, in our model the two experiences can be seen as more or less challenging ones, resembling more normally occurring environmental events during postnatal development. It is worth noting that the DER experience could be considered as analogous to the human situation where the mother is present but unavailable to the child [10].

It is worth noting that we analyzed separately the left and the right hemisphere of the rat pup brain, in order to address the issue of laterality at this neonatal stage of life, and in order to investigate the possible effects of this early experience on brain laterality. Laterality, i.e. differences in function and structure between the two brain hemispheres is quite prevalent in the animal kingdom [11], [12], [13], [14]. In the human it is particularly pronounced; the control of language being localized in the left hemisphere in more than 95% of individuals [15]. A structural corollary of this functional brain asymmetry is the finding that the left planum temporale is larger than the right [16]. The hippocampus is among the brain areas in humans showing laterality [17], a property which is of seminal importance, since abnormal left-right hippocampal asymmetry has been reported in psychopathological states, for example in schizophrenia [18], [19] and autism [20].

In the adult rat or mouse brain a number of biochemical parameters have been shown to be lateralized, such as GABA binding sites in the cerebral cortex, hippocampus, cerebellum, striatum and thalamus [21]; norepinephrine distribution in the thalamus [22]; the distribution of dopaminergic innervation [23]; expression of D2 receptors [24]; high affinity choline uptake [25]; the distribution of ε2 NMDA receptor subunits on CA1 pyramidal neurons [26]; distribution of endogenous diacylglycerol [27]; deacylation and reacylation of complex lipids [28]; protein kinase C beta II levels in the amygdala [29]. Furthermore a proteomic analysis of adult hippocampus revealed that the expression of a variety of proteins was asymmetrically distributed between the left and right hippocampi [30].

In the human, left-right brain asymmetry appears to be present early in development but the early mechanisms inducing brain laterality still remain elusive [31]. Interestingly however, Sun and co-workers [32] have shown lateralized gene transcription in the embryonic human cortex. However, in rodents, in spite of the ample evidence supporting the anatomical and biochemical left-right asymmetry of the adult brain, its emergence during development has not been clearly documented. All the above mentioned studies have been performed in the adult brain and only a few during development: Some early studies had shown that in young male rats the right hippocampus was thicker than the left, as assessed by its cross sectional width, but that this asymmetry decreased with age, and was not significantly apparent at 90 days [33]. Another recent study using transcriptomic analysis showed that there was differential gene expression between the left and right hippocampus as early as postnatal day 6, at which time point more genes were overexpressed in the right compared to the left hippocampus; interestingly, there was a right to left shift of asymmetry in gene expression during development, so that on postnatal days 9 and 60 more genes were overexpressed in the left, compared to the right hippocampus [34]. Thus, the developmental emergence of left-right brain asymmetry in the rat still remains an open question.

The effects of early experiences on brain laterality were demonstrated as early as 1978 [35], when it was shown that neonatal handling results, in adulthood, in increased sensitivity to right, compared to left, neocortical ablation. In addition early studies showed that in rats neonatal handling leads to the development of cortical laterality in emotional regulation, with the right hemisphere being more involved [36]. More recent studies have shown that handling results in increased sensitivity of the dopaminergic system in the right infralimbic cortex. [37]. Furthermore, handling and maternal separation have opposite lateralized effects on the adult medial prefrontal cortex GABA neurotransmission, the former having more pronounced effects in the right while the latter in the left hemisphere [38]. Another set of studies in which rat pups were exposed to novelty showed that this early experience altered adult hippocampal volumetric asymmetry [39], induced a shift in paw preference in a reaching task [40] and in the preference of the direction of turning during exploration of an unfamiliar environment in adulthood [41], [42], [43]. Furthermore exposure of pups to novelty increases in adulthood synaptic plasticity (short- and long-term potentiation) preferentially in the right hippocampus [44].Recently [45] maternal separation combined with early weaning (a mouse model of early neglect) has been shown to result in abnormal brain asymmetry, including that of the size of the hippocampus.

The present work is based on the hypothesis that, compared to control 13-day-old rat pups, pups exposed to either of the two early experience of our experimental model, RER and DER, would exhibit differences in protein expression in their left and right hippocampi, characteristic of the early experience. Furthermore, we hypothesized that at 13 days after birth the rat hippocampus shows left-right asymmetry in protein expression and that the early experiences could affect this laterality.

## Materials and Methods

### Ethics Statement

All animal experiments were carried out in agreement with ethical recommendation of the European Communities Council Directive of 22 September 2010 (2010/63/EU) and the experimental protocol was approved by the Ethics Committee of the Faculty of Nursing-University of Athens (# 43/9-1-09).

### Animals

Wistar rat pups born and reared in our colony were used in these experiments. Animals were kept under standard conditions (24°C, 12∶12 h light/dark cycle) and mothers received food and water *ad libitum*. Prior to the day of birth, which was designated as postnatal day 0, each litter was randomly assigned to either of the two experimental groups [pups denied (DER) or receiving (RER) the expected reward], or to the control (CTR) (non-handled) group. In this study, fifteen litters were used, five per experimental group. The average litter size [mean ± standard error of the mean (SEM): 10±1, range: 7–13] did not differ between the 3 groups. Litters were not culled, since it has been shown that litter size within this range does not affect maternal behavior [46]. The sex ratio (males/females) did not differ among the litters employed in the different animal groups [Average sex ratio (mean ± SEM): Control litters 1.07±0.10, RER litters 1.05±0.11, DER litters 1.06±0.17]. In order to maintain stable environmental stimulation for the pups, instead of cleaning the cage, wood chip was added every 4–5 days, without disturbing either the pups or the dam.

### Neonatal Training in the T-maze

The neonatal experience model has been previously described in detail [5], [6]. Pups of each litter, were trained under either Receipt of Expected Reward (RER rat pups) or Denial of Expected Reward (DER rat pups), starting from postnatal day 10 until postnatal day 13. We used a custom-made T-maze whose arms led to two cages with dimensions 30 cm width×22 cm length×30 cm height. The dimensions of the start box and the arms of the T-maze were 8 cm width×6 cm length×6 cm height and 7 cm width×30 cm length×6 cm height respectively. At the end of the one arm of the T-maze a small sliding door (9×11 cm) permitted access to the mother-containing cage when pups were trained under continuous reinforcement (RER) or remained always closed, preventing entrance into the cage, when pups were trained under the condition of denial of expected reward (DER). At the end of the other arm of the T-maze another cage was placed, without access from inside the T-maze, containing a virgin female rat for control purposes.

#### Training under Receipt of Expected Reward (RER rat pups)

Rat pups trained under the “reward” schedule were exposed to 10 trials per day (a total of 40 trials for the 4 experimental days). At the end of the duration of the trial (60 sec), or when the pup located the entrance of the mother-containing cage the sliding-door opened and the mother could retrieve the pup. If a pup did not succeed to reach the entrance of the mother-containing cage before the end of the maximum duration of the trial, it was gently guided to the entrance, the sliding door opened and the mother was allowed to retrieve the pup. In either case, following the end of the trial, the pup returned to the mother-containing cage and then the next pup was exposed to the same procedure. When all pups were exposed to the first trial the same procedure was repeated for the next trial until 10 trials were performed.

#### Training under Denial of Expected Reward (DER rat pups)

A similar training schedule was followed for the DER rats (10 trials per day for 4 training days, max duration 60 seconds each). The behavioral observation was terminated when the pup located the entrance of the mother-containing cage. If a pup did not succeed to reach the entrance of the mother-containing cage before the end of the 60 sec, it was gently guided to the entrance and had to remain there for 20 seconds. Immediately after the end of the trial the pup was returned to the start box of the T-maze for the next trial. Following the completion of 10 trials the pup was returned to the mother-containing cage and then another pup was exposed to the same procedure. At the end of the 10 daily trials for all littermates, the mother, followed by the litter, was returned to the home cage in the animal facility room.

### Tissue Preparation and 2-dimensional Electrophoresis (2-DE) Gels

On postnatal day 13, two hours after their exposure to the last training trial, pups were euthanized, decapitated, brains were removed and the left and right hippocampus were dissected. Dissected hippocampi were stored at −80°C until further processing. For each experimental group (RER, CTR, DER) 2 samples were prepared, in each of which 5, either left or right, hippocampi were pooled. Tissue homogenization was performed as previously described [30], [47]: Hippocampi were suspended in 350 µl sample buffer containing 20 mM Tris, 7 M urea, 2 M thiourea, 4% CHAPS, 10 mM 1,4-dithioerythritol, 1 mM EDTA, and a mixture of protease inhibitors (1 mM PMSF and 1 tablet CompleteTM (Roche Diagnostics, Basel, Swiss) per 50 ml of suspension buffer) and phosphatase inhibitors (0.2 mM Na_2_VO_3_ and 1 mM NaF). The suspension was sonicated for ∼30 s and centrifuged at 150,000 g for 20 min. The protein content in the supernatant was determined using the Bradford method.

For the isoelectric focusing, 1 mg total protein was applied by the non-cup technique on 17 cm immobilized pH 3–10 nonlinear gradient IPG strips (Bio-Rad, Hercules, CA,USA) and electrophoresed for 90 kVh. For the second-dimension of electrophoresis 12% sodium dodecyl sulfate (SDS) polyacrylamide gels were run in a Proteiner apparatus (Bio-Rad) and the gels were stained with colloidal Coomassie Blue (Novex, San Diego, CA,USA). The six samples (right-left hippocampi×3 experimental groups) were subjected to 2-D electrophoresis concomitantly, while the other six (biological replicate) were run (together) at a different time, as a separate analysis.

### Gel Image Analysis

Coomassie stained gels were scanned on a GS-800 BioRad calibrated densitometer. The resulting TIFF images were analyzed using the PDQuest Software v. 8.0. Protein spots showing differences in their expression level were identified and mean spot volume was calculated. In the final tables only proteins showing at least a 2-fold (200%) difference in expression level were included.

### Matrix-Assisted Laser Desorption/Ionization Time-of-Flight Mass Spectrometry *(MALDI-TOF-MS)*

Peptide analysis and protein identification were performed as previously described [30], [47]. The selected spots were manually detected on the gels by Melanie 4.02 software (GeneBio, Geneve Bioinformatics S.A., Geneva, Swiss). They were excised by the Proteiner SPII (Bruker Daltonics, Bremen, Germany), destained with 30% acetonitrile in 50 mM ammonium bicarbonate, dried in a speed vacuum concentrator (MaxiDry Plus; Heto, Allered, Denmark) and rehydrated with 5 µl of 1 mM ammonium bicarbonate containing 50 ng trypsin (Roche Diagnostics) and left in the dark overnight at room temperature. Twenty microlitre of 50% acetonitrile, containing 0.1% trifluoroacetic acid were added to each gel piece and incubated for 15 min with constant shaking. The resulting peptide mixture (1 µl) was simultaneously applied with 1 µl of matrix solution, consisting of 0.8% a-cyano-4-hydroxycinnamic acid (Sigma-Aldrich), standard peptides des-Arg-bradykinin (904.4681 Da; Sigma -Aldrich), and adrenocorticotropic hormone fragment 18–39, (2465.1989 Da; Sigma-Aldrich) in 50% acetonitrile and 0.1% trifluoroacetic acid. Samples were analyzed with matrix-assisted laser desorption-mass spectrometry (MALDI-MS) in a time-of-flight mass spectrometer (Ultraflex II, Bruker Daltonics, Bremen, Germany). Laser shots (n  = 1,000) at intensity between 40 and 60% were collected and summarized using the FlexControl v2.2 software by Bruker. Peak list was created with Flexanalysis v2.2 software. Smoothing was applied with the Savitzky-Golay algorithm (width 0.2 mz, cycle number 1). S/N threshold ratio of 2.5 was allowed. SNAP algorithm was used for peak picking. Matching peptides and protein searches were performed automatically. Each spectrum was interpreted with the Mascot Software v. 2.0 (Matrix Sciences, London, UK) which is a software search engine that uses mass spectrometry data to identify proteins from primary sequence database. This software compares the mass spectrometry data obtained experimentally, to all sequences in a database and provides a list of hits with decreasing scores which represent the degree of the reliability of identification: The higher the score, the better the identification. In practice, positive identification of a protein is accepted if the mascot score obtained for this protein is higher than the set threshold [48], [49]. In our study, the mascot score was set at >49 (p<0.05), which corresponds to a probability of 95% that the protein identified is not a random match. For peptide identification, the monoisotopic masses were used and a mass tolerance of 0.0025% (25 ppm) was allowed. All extraneous peaks, such as trypsin autodigests, matrix, and keratin peaks, were not considered in the protein search. Cysteine carbamidomethylation and methionine oxidation were set as fixed and variable modifications, respectively. One miscleavage was allowed. The peptide masses were then compared with the theoretical peptide masses of all proteins from rodents using the SWISS-PROT and TREMBL protein sequence databases.

### Western Blotting

For selected proteins differences in expression level detected by the proteomics analysis were confirmed by western blotting. For each group (RER, DER CTR) the left and right hippocampus of 3 different non-littermate 13-day-old pups was homogenized separately and subjected to western analysis, performed as previously described [7]. It should be noted that the 3 animals per group used for Western blotting were different from the ones used in the proteomic analysis. Each hippocampus, right or left, was homogenized in 120 µl of an ice cold lysis buffer (20 mM Tris HCl pH 7.6, 137 mM NaCl, 48 mM NaF, 2 mM Na_3_VO_4,_ 1% SDS, and 10% glycerol, 1∶250 Protease Inhibitor Cocktail Sigma). The homogenates were centrifuged at 14,000 rpm for 20 min at 4°C. Hippocampal protein lysates were resolved on 4–12% NuPAGE® Bis-Tris precast polyacrylamide gels (Invitrogen, USA), and blotted to 0.45 µm nitrocellulose membranes (Whatman, Schleicher and Schuel; Dassel, Germany). In order to prevent non-specific staining membranes were blocked for 2 h at room temperature with 5% nonfat dry milk in TBS containing 0.05% Tween-20. The membranes were then incubated overnight with primary antibodies and afterwards with the appropriate secondary HRP-conjugated antibodies (from Santa Cruz and Millipore) in TBST. The primary antibodies used were anti-glyceraldehydes-3-phosphate dehydrogenase (GAPDH) mouse monoclonal antibody 1∶1000 (MAB374 Millipore, Billenica, MA, USA), anti-GFAP 1∶1000 (Z334, DakoCytomation), anti-heat-shock protein 70 (HSP70) 1∶1000 (sc-1060, Santa Cruz Biotechnology Inc., CA, USA), anti-dihydropyrimidinase like protein 1 (DRP1) 1∶2000 (Ab36191, Abcam), anti-tubulin beta 1∶2000 (T4026, Sigma-Aldrich, St. Louis, MO, USA), anti-β-actin 1∶1000 (sc-47778, Santa Cruz Biotechnology Inc., CA, USA). The signal was visualized on autoradiographic films (Kodak XAR) by chemiluminescence (Amersham, UK). Quantification of the amount of each protein was performed using the Image J Software. The optical density of each band was divided by the optical density of the respective GAPDH band, since GAPDH expression did not show any differences between groups or right and left hippocampi, in our proteomic analysis.

### Statistical Analysis

Data from Western blots were analyzed by one-way repeated measures ANOVA with the side of the brain as the repeated measure and the experimental group (control, RER, DER) as the independent factor.

## Results

**Figure 1:**
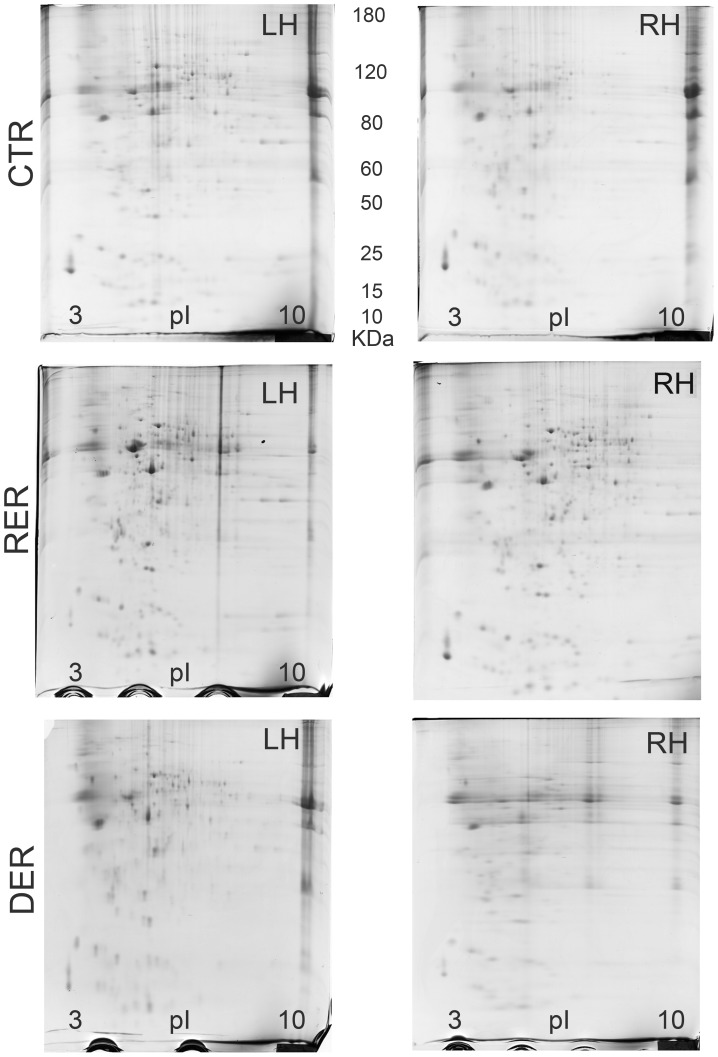
Representative 2-DE gels of 13-day-old CTR, RER, DER pup left (LH) and right (RH) hippocampus.

The PD-Quest 2D analysis software demonstrated that using a broad range pH 3–10 IPG strip for the first dimension, resulted in the appearance of a large number of spots in each gel. See Figure 1 for representative gels of the proteins in the right and left hippocampus of 13-day-old CTR, RER, and DER rat pups. We identified and spot-picked a total of 414 spots which were differentially expressed either between experimental groups, or right and left hippocampi. These spots were then gel-excised and trypsin-digested and the trypsin digests were analyzed by mass spectrometry. We, thus, identified 96 different proteins. Tables 1, 2, 3, 4, 5, 6, 7, 8, 9, 10, 11, 12, 13, 14, 15, 16, 17, 18 show the differences in protein levels in either the left or the right hippocampus between the three experimental groups, or between the left and right hippocampus within each experimental group. Only proteins which showed at least a 2-fold difference in both replicates of any of the above mentioned comparisons were included in the tables.

**Table 1:** Proteins (5) with higher levels in the Left Hippocampus (LH) of the DER compared to those of the CTR.

Accession number	Protein ID fromSwiss Prot	Protein name	Mascot Score	Levels in DERLevels in CTR
P10719	ATPB_RAT	ATP synthase subunit beta, mitochondrial	202	12
P10860	DHE3_RAT	Glutamate dehydrogenase 1, mitochondrial	115	892
P04764	ENOA_RAT	Alpha-enolase	197	6492
P07323	ENOG_RAT	Gamma-enolase	217	1520
O88767	PARK7_RAT	Protein DJ-1	62	18

**Table 2:** Proteins (3) with higher levels in the Left Hippocampus (LH) of the CTR compared to those of the DER.

Accession number	Protein ID fromSwiss Prot	Protein name	Masco Score	Levels in CTRLevels in DER
Q62951	DPYL4_RAT	Dihydropyrimidinase-related protein	177	4
P16086	SPTA2_RAT	Spectrin alpha chain, brain	232	15
Q6P9T8	TBB2C_RAT	Tubulin beta-2C chain	57	15

**Table 3:** Proteins (17) with higher levels in the Left Hippocampus (LH) of the RER compared to those of the CTR.

Accessionnumber	Protein ID fromSwiss Prot	Protein name	MascotScore	Levels in RERLevels in CTR
Q9ER34	ACON_RAT	Aconitate hydratase, mitochondrial	79	383
P23565	AINX_RAT	Alpha-internexin	56	8
P02770	ALBU_RAT	Serum albumin	157	616
P07323	ENOG_RAT	Gamma-enolase	204	9
Q66HD0	ENPL_RAT	Endoplasmin	119	68
P85845	FSCN1_RAT	Fascin	94	6
Q5XI73	GDIR1_RAT	Rho GDP-dissociation inhibitor 1	99	816
P48721	GRP75_RAT	Stress-70 protein, mitochondrial	111	1994
P06761	GRP78_RAT	78 kDa glucose-regulated protein	242	7
P34058	HS90B_RAT	Heat shock protein HSP 90-beta	146	820
O88600	HSP74_RAT	Heat shock 70 kDa protein 4	235	23
P19527	NFL_RAT	Neurofilament light polypeptide	174	8
O88767	PARK7_RAT	Protein DJ-1	54	15
P31044	PEBP1_RAT	Phosphatidylethanolamine-binding protein 1	110	5
P25113	PGAM1_RAT	Phosphoglycerate mutase 1	160	3
P46462	TERA_RAT	Transitional endoplasmic reticulum ATPase	252	3
Q00981	UCHL1_RAT	Ubiquitin carboxyl-terminal hydrolase isozyme L1	142	11249

**Table 4:** Proteins (7) with higher levels in the Left Hippocampus (LH) of the CTR compared to those of the RER.

Accession number	Protein ID from Swiss Prot	Protein name	MascotScore	Levels in CTRLevels in RER
P60711	ACTB_RAT	Actin, cytoplasmic 1	116	7
P63259	ACTG_RAT	Actin, cytoplasmic 2	108	9
P10719	ATPB_RAT	ATP synthase subunit beta, mitochondrial	149	3
P47942	DPYL2_RAT	Dihydropyrimidinase-related protein 2	64	8
P47819	GFAP_RAT	Glial fibrillary acidic protein	243	7
P63018	HSP7C_RAT	Heat shock cognate 71 kDa protein	194	25620
P08461	ODP2_RAT	Dihydrolipoyllysine-residue acetyltransferase componentof pyruvate dehydrogenase complex	64	4

**Table 5:** Proteins (15) with higher levels in the Left Hippocampus (LH) of the DER compared to those of the RER.

Accessionnumber	Protein ID from Swiss Prot	Protein name	MascotScore	Levels in DERLevels in RER
P23565	AINX_RAT	Alpha-internexin	220	5
P47942	DPYL2_RAT	Dihydropyrimidinase-related protein 2	171	3
Q62952	DPYL3_RAT	Dihydropyrimidinase-related protein 3	89	3
P04764	ENOA_RAT	Alpha-enolase	197	14
P63018	HSP7C_RAT	Heat shock cognate 71 kDa protein	226	14611
P11598	PDIA3_RAT	Protein disulfide-isomerase A3	112	4
Q6P9V9	TBA1B_RAT	Tubulin alpha-1B chain	144	87
Q6AYZ1	TBA1C_RAT	Tubulin alpha-1C chain	112	14
Q68FR8	TBA3_RAT	Tubulin alpha-3 chain	121	87
Q5XIF6	TBA4A_RAT	Tubulin alpha-4A chain	121	87
P85108	TBB2A_RAT	Tubulin beta-2A chain	180	13
Q3KRE8	TBB2B_RAT	Tubulin beta-2B chain	214	10
Q6P9T8	TBB2C_RAT	Tubulin beta-2C chain	163	10
Q4QRB4	TBB3_RAT	Tubulin beta-3 chain	141	13
P69897	TBB5_RAT	Tubulin beta-5 chain	229	10

**Table 6:** Proteins (14) with higher levels in the Left Hippocampus (LH) of the RER compared to those of the DER.

Accessionnumber	Protein ID from Swiss Prot	Protein name	Mascot Score	Levels in RERLevels in DER
P63102	1433Z_RAT	14-3-3 protein zeta/delta	124	3370
P02770	ALBU_RAT	Serum albumin	157	4
Q3T1K5	CAZA2_RAT	F-actin-capping protein subunit alpha-2	72	1522
B0BNA5	COTL1_RAT	Coactosin-like protein	71	34
Q62951	DPYL4_RAT	Dihydropyrimidinase-related protein 4	177	11
P50398	GDIA_RAT	Rab GDP dissociation inhibitor alpha	199	7510
P47819	GFAP_RAT	Glial fibrillary acidic protein	237	2084
O88600	HSP74_RAT	Heat shock 70 kDa protein 4	235	6
Q6P7Q4	LGUL_RAT	Lactoylglutathione lyase	104	10
P08461	ODP2_RAT	Dihydrolipoyllysine-residue acetyltransferase componentof pyruvate dehydrogenase complex	64	7
P38983	RSSA_RAT	40S ribosomal protein SA	118	3
Q6AY84	SCRN1_RAT	Secernin-1	121	77
P16086	SPTA2_RAT	Spectrin alpha chain, brain	56	4
P46462	TERA_RAT	Transitional endoplasmic reticulum ATPase	252	3

**Table 7:** Proteins (4) with higher levels in the Right Hippocampus (RH) of the DER compared to those of the CTR.

Accessionnumber	Protein ID fromSwiss Prot	Protein name	Mascot Score	Levels in DERLevels in CTR
P04764	ENOA_RAT	Alpha-enolase	107	3
P63018	HSP7C_RAT	Heat shock cognate 71 kDa protein	83	8
P46462	TERA_RAT	Transitional endoplasmic reticulum ATPase	269	211
P85972	VINC_RAT	Vinculin	103	1088

**Table 8:** Proteins (25) with higher levels in the Right Hippocampus (RH) of the CTR compared to those of the DER.

Accessionnumber	Protein ID fromSwiss Prot	Protein name	Mascot Score	Levels in CTRLevels in DER
P63039	CH60_RAT	60 kDa heat shock protein, mitochondrial	157	1417
P51635	AK1A1_RAT	Alcohol dehydrogenase [NADP+]	96	238
P10719	ATPB_RAT	ATP synthase subunit beta, mitochondrial	173	2
P31399	ATP5H_RAT	ATP synthase subunit d, mitochondrial	110	3
Q6P6R2	DLDH_RAT	Dihydrolipoyl dehydrogenase, mitochondrial	92	355
Q62950	DPYL1_RAT	Dihydropyrimidinase-related protein 1	141	4
P47942	DPYL2_RAT	Dihydropyrimidinase-related protein 2	133	538
Q66HD0	ENPL_RAT	Endoplasmin	105	5
P09117	ALDOC_RAT	Fructose-bisphosphate aldolase C	197	2
P07323	ENOG_RAT	Gamma-enolase	209	4
P62994	GRB2_RAT	Growth factor receptor-bound protein 2	91	5
P42123	LDHB_RAT	L-lactate dehydrogenase B chain	100	1945
O35244	PRDX6_RAT	Peroxiredoxin-6	180	5
P31044	PEBP1_RAT	Phosphatidylethanolamine-binding protein 1	123	2
P25113	PGAM1_RAT	Phosphoglycerate mutase 1	136	1179
P48721	GRP75_RAT	Stress-70 protein, mitochondrial	233	3
Q5XIM9	TCPB_RAT	T-complex protein 1 subunit beta	83	850
P48500	TPIS_RAT	Triosephosphate isomerase	167	155
P68370	TBA1A_RAT	Tubulin alpha-1A chain	177	4
Q6P9V9	TBA1B_RAT	Tubulin alpha-1B chain	177	4
Q6AYZ1	TBA1C_RAT	Tubulin alpha-1C chain	142	4
Q68FR8	TBA3_RAT	Tubulin alpha-3 chain	132	4
Q5XIF6	TBA4A_RAT	Tubulin alpha-4A chain	145	4
Q00981	UCHL1_RAT	Ubiquitin carboxyl-terminal hydrolase isozyme L1	107	4
Q4KM73	KCY_RAT	UMP-CMP kinase	146	9

**Table 9:** Proteins (16) with higher levels in the Right Hippocampus (RH) of the RER compared to those of the CTR.

Accession number	Protein ID fromSwiss Prot	Protein name	Mascot Score	Levels in RERLevels in CTR
P60711	ACTB_RAT	Actin, cytoplasmic 1	89	22
P63259	ACTG_RAT	Actin, cytoplasmic 2	89	22
P38062	AMPM2_RAT	Methionine aminopeptidase 2	105	2
P18418	CALR_RAT	Calreticulin	235	6
Q62952	DPYL3_RAT	Dihydropyrimidinase-related protein 3	175	3
P04764	ENOA_RAT	Alpha-enolase	107	2
P54311	GBB1_RAT	Guanine nucleotide-binding protein G(I)/G(S)/G(T) subunit beta-1	127	2396
P54313	GBB2_RAT	Guanine nucleotide-binding protein G(I)/G(S)/G(T) subunit beta-2	70	2396
P47819	GFAP_RAT	Glial fibrillary acidic protein	358	3
Q66HF1	NDUS1_RAT	NADH-ubiquinone oxidoreductase 75 kDa subunit, mitochondrial	163	2
P19527	NFL_RAT	Neurofilament light polypeptide	126	626
O35987	NSF1C_RAT	NSFL1 cofactor p47	54	22
P08461	ODP2_RAT	Dihydrolipoyllysine-residue acetyltransferase component of pyruvate dehydrogenase complex	118	2
P11598	PDIA3_RAT	Protein disulfide-isomerase A3	195	3
P46462	TERA_RAT	Transitional endoplasmic reticulum ATPase	269	778
P85972	VINC_RAT	Vinculin	139	3

**Table 10:** Proteins (9) with higher levels in the Right Hippocampus (RH) of the CTR compared to those of the RER.

Accession number	Protein ID fromSwiss Prot	Protein name	MascotScore	Levels in CTRLevels in RER
Q6P6R2	DLDH_RAT	Dihydrolipoyl dehydrogenase, mitochondrial	92	488
P48721	GRP75_RAT	Stress-70 protein, mitochondrial	233	968
Q99NA5	IDH3A_RAT	Isocitrate dehydrogenase [NAD] subunit alpha, mitochondrial	167	2
Q05982	NDKA_RAT	Nucleoside diphosphate kinase A	97	2
P07632	SODC_RAT	Superoxide dismutase [Cu-Zn]	76	1406
P85108	TBB2A_RAT	Tubulin beta-2A chain	197	8
Q6P9T8	TBB2C_RAT	Tubulin beta-2C chain	150	8
Q4QRB4	TBB3_RAT	Tubulin beta-3 chain	121	8
P69897	TBB5_RAT	Tubulin beta-5 chain	197	8

**Table 11:** Protein (1) with higher levels in the Right Hippocampus (RH) of the DER compared to those of the RER.

Accession number	Protein ID fromSwiss Prot	Protein name	Mascot Score	Levels in DERLevels in RER
P63018	HSP7C_RAT	Heat shock cognate 71 kDa protein	83	2

**Table 12:** Proteins (27) with higher levels in the Right Hippocampus (RH) of the RER compared to those of the DER.

Accession number	Protein ID fromSwiss Prot	Protein name	MascotScore	Levels in RERLevels in DER
Q9ER34	ACON_RAT	Aconitate hydratase, mitochondrial	283	4
P60711	ACTB_RAT	Actin, cytoplasmic 1	126	3
P63259	ACTG_RAT	Actin, cytoplasmic 2	126	3
P10719	ATPB_RAT	ATP synthase subunit beta, mitochondrial	226	3
Q3T1K5	CAZA2_RAT	F-actin-capping protein subunit alpha-2	64	3
P10860	DHE3_RAT	Glutamate dehydrogenase 1, mitochondrial	172	3
Q62950	DPYL1_RAT	Dihydropyrimidinase-related protein 1	74	2
P47942	DPYL2_RAT	Dihydropyrimidinase-related protein 2	222	935
Q62951	DPYL4_RAT	Dihydropyrimidinase-related protein 4	135	3
Q9JHU0	DPYL5_RAT	Dihydropyrimidinase-related protein 5	107	3
P07323	ENOG_RAT	Gamma-enolase	220	5
Q5XI73	GDIR1_RAT	Rho GDP-dissociation inhibitor 1	96	38
Q66HA8	HS105_RAT	Heat shock protein 105 kDa	99	4
O88600	HSP74_RAT	Heat shock 70 kDa protein 4	147	4
P07335	KCRB_RAT	Creatine kinase B-type	176	16
P11980	KPYM_RAT	Pyruvate kinase isozymes M1/M2	214	10
P42123	LDHB_RAT	L-lactate dehydrogenase B chain	170	3
O88989	MDHC_RAT	Malate dehydrogenase, cytoplasmic	67	3
Q05982	NDKA_RAT	Nucleoside diphosphate kinase A	94	183
P19527	NFL_RAT	Neurofilament light polypeptide	126	585
Q9QUL6	NSF_RAT	Vesicle-fusing ATPase	96	1870
P31044	PEBP1_RAT	Phosphatidylethanolamine-binding protein 1	55	2
O35567	PUR9_RAT	Bifunctional purine biosynthesis protein PURH	53	437
P38983	RSSA_RAT	40S ribosomal protein SA	176	1436
B2GV06	SCOT1_RAT	Succinyl-CoA:3-ketoacid-coenzyme A transferase 1, mitochondrial	109	2
P46462	TERA_RAT	Transitional endoplasmic reticulum ATPase	214	3
Q00981	UCHL1_RAT	Ubiquitin carboxyl-terminal hydrolase isozyme L1	126	4

**Table 13:** Proteins (25) with higher levels in the Left Hippocampus (LH) compared to those in the Right Hippocampus (RH) of the CTR.

Accession number	Protein ID fromSwiss Prot	Protein name	Mascot Score	Levels in LH of the CTRLevels in RH of the CTR
Q9ER34	ACON_RAT	Aconitate hydratase, mitochondrial	95	730
P60711	ACTB_RAT	Actin, cytoplasmic 1*	109	18
P63259	ACTG_RAT	Actin, cytoplasmic 2	109	18
P23565	AINX_RAT	Alpha-internexin*	243	22
P62161	CALM_RAT	Calmodulin	71	2756
Q91ZN1	COR1A_RAT	Coronin-1A	113	5820
P10860	DHE3_RAT	Glutamate dehydrogenase 1, mitochondrial	153	10
Q62952	DPYL3_RAT	Dihydropyrimidinase-related protein 3*	161	20
P54311	GBB1_RAT	Guanine nucleotide-binding protein G(I)/G(S)/G(T) subunit beta*	65	146
P82995	HS90A_RAT	Heat shock protein HSP 90-alpha	82	1097
P34058	HS90B_RAT	Heat shock protein HSP 90-beta	121	1097
P63018	HSP7C_RAT	Heat shock cognate 71 kDa protein	63	31
P07335	KCRB_RAT	Creatine kinase B-type*	58	73809
P11598	PDIA3_RAT	Protein disulfide-isomerase A3	167	21
P38983	RSSA_RAT	40S ribosomal protein SA	121	5
B2GV06	SCOT1_RAT	Succinyl-CoA:3-ketoacid-coenzyme A transferase 1, mitochondrial	58	19
P68370	TBA1A_RAT	Tubulin alpha-1A chain*	175	6
Q6P9V9	TBA1B_RAT	Tubulin alpha-1B chain*	103	20
Q6AYZ1	TBA1C_RAT	Tubulin alpha-1C chain	68	146
Q68FR8	TBA3_RAT	Tubulin alpha-3 chain	77	20
Q5XIF6	TBA4A_RAT	Tubulin alpha-4A chain	54	19
Q5XIM9	TCPB_RAT	T-complex protein 1 subunit beta	132	4
P46462	TERA_RAT	Transitional endoplasmic reticulum ATPase	269	513
Q63610	TPM3_RAT	Tropomyosin alpha-3 chain	91	3
P62815	VATB2_RAT	V-type proton ATPase subunit B, brain isoform*	235	6

* Proteins showing reversed asymmetry of expression (right over left) in the adult hippocampus.

**Table 14:** Proteins (7) with higher levels in the Right Hippocampus (RH) compared to those in the Left Hippocampus (LH) of the CTR.

Accession number	Protein ID fromSwiss Prot	Protein name	Mascot Score	Levels in RH of the CTRLevels in LH of the CTR
P62161	CALM_RAT	Calmodulin	85	11
Q62950	DPYL1_RAT	Dihydropyrimidinase-related protein 1	141	5
P85845	FSCN1_RAT	Fascin	183	828
Q5XI73	GDIR1_RAT	Rho GDP-dissociation inhibitor 1	137	12
Q6DGG0	PPID_RAT	40 kDa peptidyl-prolyl cis-trans isomerase	72	3
P35704	PRDX2_RAT	Peroxiredoxin-2	54	4
P31000	VIME_RAT	Vimentin	206	3

**Table 15:** Proteins (24) with higher levels in the Left Hippocampus (LH) compared to those in the Right Hippocampus (RH) of the DER.

Accession number	Protein ID fromSwiss Prot	Protein name	MascotScore	Levels in LH of the DERLevels in RH of the DER
P60711	ACTB_RAT	Actin, cytoplasmic 1	70	2273
P63259	ACTG_RAT	Actin, cytoplasmic 2	70	2273
Q9ER34	ACON_RAT	Aconitate hydratase, mitochondrial	56	128
Q6P6R2	DLDH_RAT	Dihydrolipoyl dehydrogenase, mitochondrial	54	5
Q62952	DPYL3_RAT	Dihydropyrimidinase-related protein 3	89	5
P85834	EFTU_RAT	Elongation factor Tu, mitochondrial	118	594
P63018	HSP7C_RAT	Heat shock cognate 71 kDa protein	231	941
P19527	NFL_RAT	Neurofilament light polypeptide	107	3
P12839	NFM_RAT	Neurofilament medium polypeptide	173	4
P11598	PDIA3_RAT	Protein disulfide-isomerase A3	112	20
B2GV06	SCOT1_RAT	Succinyl-CoA:3-ketoacid-coenzyme A transferase 1, mitochondrial	61	489
O35814	STIP1_RAT	Stress-induced-phosphoprotein 1	115	6
P16086	SPTA2_RAT	Spectrin alpha chain, brain	232	785
P68370	TBA1A_RAT	Tubulin alpha-1A chain	173	76
Q6P9V9	TBA1B_RAT	Tubulin alpha-1B chain	173	76
Q6AYZ1	TBA1C_RAT	Tubulin alpha-1C chain	139	76
Q68FR8	TBA3_RAT	Tubulin alpha-3 chain	126	76
Q5XIF6	TBA4A_RAT	Tubulin alpha-4A chain	141	76
P85108	TBB2A_RAT	Tubulin beta-2A chain	191	242
Q3KRE8	TBB2B_RAT	Tubulin beta-2B chain	191	242
Q6P9T8	TBB2C_RAT	Tubulin beta-2C chain	142	242
Q4QRB4	TBB3_RAT	Tubulin beta-3 chain	97	242
P69897	TBB5_RAT	Tubulin beta-5 chain	174	242

**Table 16:** Proteins (7) with higher levels in the Right Hippocampus (RH) compared to those in the Left Hippocampus (LH) of the DER.

Accession number	Protein ID fromSwiss Prot	Protein name	Mascot Score	Levels in RH of the DERLevels in LH of the DER
P62161	CALM_RAT	Calmodulin	77	4
Q62951	DPYL4_RAT	Dihydropyrimidinase-related protein 4	179	1605
Q9JHU0	DPYL5_RAT	Dihydropyrimidinase-related protein 5	135	1605
P31977	EZRI_RAT	Ezrin	67	8
O88600	HSP74_RAT	Heat shock 70 kDa protein 4	101	108
O88989	MDHC_RAT	Malate dehydrogenase, cytoplasmic	82	2
P85972	VINC_RAT	Vinculin	103	6

**Table 17:** Proteins (18) with higher levels in the Left Hippocampus (LH) compared to those in the Right Hippocampus (RH) of the RER.

Accession number	Protein ID fromSwiss Prot	Protein name	MascotScore	Levels in LH of the RERLevels in RH of the RER
P23565	AINX_RAT	Alpha-internexin	220	20
P18418	CALR_RAT	Calreticulin	166	16
B0BNA5	COTL1_RAT	Coactosin-like protein	71	3
P85845	FSCN1_RAT	Fascin	163	2
P54311	GBB1_RAT	Guanine nucleotide-binding protein G(I)/G(S)/G(T) subunit beta	75	6667
P50398	GDIA_RAT	Rab GDP dissociation inhibitor alpha	199	3755
P82995	HS90A_RAT	Heat shock protein HSP 90-alpha	66	4
P34058	HS90B_RAT	Heat shock protein HSP 90-beta	105	4
P63018	HSP7C_RAT	Heat shock cognate 71 kDa protein	90	4
P19527	NFL_RAT	Neurofilament light polypeptide	174	8
Q6AY84	SCRN1_RAT	Secernin-1	94	109
P16086	SPTA2_RAT	Spectrin alpha chain, brain	56	370
P68370	TBA1A_RAT	Tubulin alpha-1A chain	98	2
Q6P9V9	TBA1B_RAT	Tubulin alpha-1B chain	160	16536
Q6AYZ1	TBA1C_RAT	Tubulin alpha-1C chain	128	16536
Q68FR8	TBA3_RAT	Tubulin alpha-3 chain	117	16536
Q5XIF6	TBA4A_RAT	Tubulin alpha-4A chain	145	16536
Q68FQ0	TCPE_RAT	T-complex protein 1 subunit epsilon	71	1986

**Table 18:** Proteins (10) with higher levels in the Right Hippocampus (RH) compared to those in the Left Hippocampus (LH) of the RER.

Accession number	Protein ID fromSwiss Prot	Protein name	Mascot Score	Levels in RH of the RERLevels in LH of the RER
Q05175	BASP1_RAT	Brain acid soluble protein 1	58	52
P86182	CCD22_RAT	Coiled-coil domain-containing protein 22	61	8
P63039	CH60_RAT	60 kDa heat shock protein, mitochondrial	112	8
P08082	CLCB_RAT	Clathrin light chain B	78	2
Q62950	DPYL1_RAT	Dihydropyrimidinase-related protein 1	138	4
Q62951	DPYL4_RAT	Dihydropyrimidinase-related protein 4	192	9
Q9JHU0	DPYL5_RAT	Dihydropyrimidinase-related protein 5	130	9
P31977	EZRI_RAT	Ezrin	62	2
P08461	ODP2_RAT	Dihydrolipoyllysine-residue acetyltransferase component of pyruvate dehydrogenase complex	71	4
P85972	VINC_RAT	Vinculin	70	117

### Group Differences

Tables 1, 2, 3, 4, 5, 6 present the proteins which have a different expression profile among the three groups (CTR, RER, DER) in the left hippocampus and Tables 7, 8, 9, 10, 11, 12 show the hippocampal proteins whose expression differs among the three groups in the right hippocampus.

Generally, the number of proteins whose expression was up-regulated (in- comparison to the CTR group) as a result of the early experience was greater in the RER animals than in the DER. In both the left and right hippocampus, in comparison to the CTR group, the RER animals had more up-regulated proteins than did the DER. More specifically, in the left hippocampus the RER animals had 17 up-regulated proteins while the DER only 5. Similarly, in the right hippocampus the RER animals had 16 up-regulated proteins, in comparison to the CTR, while the DER had only 4 such proteins. Interestingly, in the left hippocampus, the RER experience resulted not only in increased up-regulation, but also in increased down-regulation of protein expression: In the left hippocampus when compared to the CTR, the RER animals had a greater number of down-regulated proteins (7) than did the DER (3). In contrast, in the right hippocampus the DER - compared to the CTR-had a higher number of down-regulated proteins (25) than did the RER (9).

The comparison between the RER and DER groups of animals revealed that the two early experiences did not differ between them as to the number of proteins whose expression was altered (up- or down-regulated) in the left hippocampus. Of course there were qualitative differences, since the expression of different proteins was affected in the RER and the DER. In contrast, in the right hippocampus, only one protein, heat shock cognate protein 71, was up-regulated while 27 proteins were down-regulated in the DER compared to the RER.

### Laterality

Interestingly, proteomic analysis showed that more proteins had higher levels in the left than the right hippocampus of the 13-day-old CTR rat pups, while for fewer proteins the opposite held true. Table 13 shows the proteins that were found to have higher levels in the left hippocampus compared to the right, while Table 14 shows the proteins whose levels were higher in the right hippocampus compared to the left. More specifically, there were 25 proteins (5 of them were tubulins, 20 non-tubulins) that had higher levels in the left side, while only 7 proteins (all of them were non-tubulins) were found to have higher levels in the right hippocampus. Moreover, it was quite interesting that the group of animals which were Denied the Expected Reward (DER rat pups) followed the same pattern of left-right hippocampal asymmetry regarding protein up-regulation as the CTR group. Thus, it was found that in the left hippocampus of the DER rat pups there were 24 proteins (10 belonged to the tubulin family of proteins, 14 were non-tubulins) with higher levels in the left than the right, while only 7 proteins (all of them were non-tubulins) had higher levels in the right than the left hippocampus. The proteins whose levels were higher in the left or right hippocampus of the DER rat pups are shown in tables 15 and 16 respectively. Left-right asymmetry in the RER group appeared to be less pronounced than in the control or DER group. More specifically, only 18 proteins (5 of them were tubulins, and 13 were non-tubulins) were found to have higher levels in the left than the right hippocampus, while 10 (all of them being non-tubulin proteins) were up-regulated in the right compared to the left hippocampus. Table 17 presents the proteins whose levels were higher in the left hippocampus compared to the right, and Table 18 the proteins whose levels were higher in the right hippocampus compared to the left of the RER rat pups. In all three experimental groups (CTR, RER, DER) tubulin had higher levels in the left than the right hippocampus.

### Confirmation of the Group Differences and Laterality Results by Western Blotting

**Figure 2:**
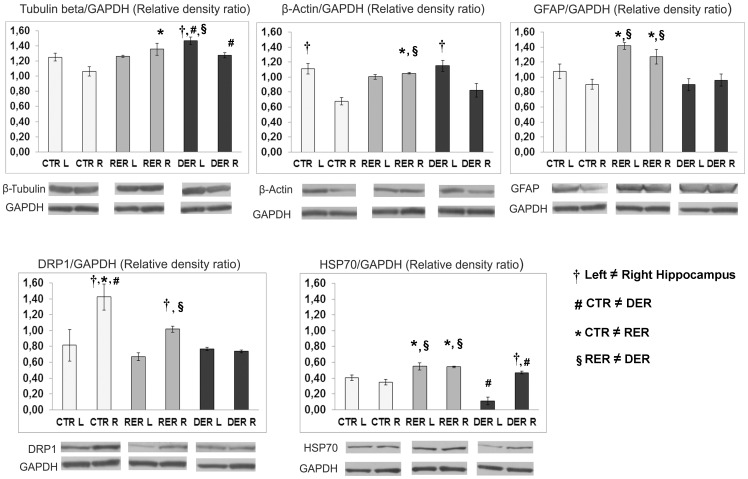
Western blot analysis of selected proteins found by the proteomic analysis to be differentially expressed. Bars represent normalized (vs GAPDH) protein levels (Mean ± S.E.M.). Symbols denote statistically significant differences.

Immunoblotting was used to confirm some of the protein expression differences detected through the differential proteomics approach. Protein lysates from left and right hippocampus from individual animals were resolved by gel electrophoresis and blotted with the appropriate antibody (as described in the Materials and Methods Section). Figure 2 shows representative western blot analyses of some of the proteins found by the proteomic analysis to differ either between experimental groups or between left and right hippocampi. These were β-tubulin, β-actin, glial fibrillary acidic protein (GFAP), dihydropyrimidinase like protein 1 (DRP1), heat-shock protein 70 (HSP70). The bars correspond to the relative quantification of the proteins, using GAPDH as the gel loading control.

Statistical analysis using one way repeated measures ANOVA with the side of the brain as the repeated measure and the group of animals (CTR, RER, DER) as the independent factor for β-tubulin levels revealed a statistically significant side × group interaction (F_1,6_ = 7.896, p = 0.021). Post hoc analysis indicated that in the DER group higher levels of β-tubulin were found in the left than in the right hippocampus († p = 0.041). Moreover, in the right hippocampus control animals had lower levels of β-tubulin compared to either the RER or the DER animals (post hoc, *p = 0.006 CTR vs. RER, # p = 0.025 CTR vs. DER), while in the left hippocampus both the control and the RER animals had lower levels than the DER (post hoc, # p = 0.004 CTR vs. DER, § p = 0.004 RER vs. DER).

Similar analysis showed for β-actin protein levels a statistically significant side × group interaction (F_1,6_ = 23.471, p = 0.001). Further analysis indicated that in both the control and the DER group β-actin levels were higher in the left than in the right hippocampus († p<0.05 for both groups) while no laterality was observed in the RER group. In addition, in the right hippocampus RER animals had higher levels of β-actin compared to both the CTR and the DER (pos hoc, *p = 0.008 CTR vs. RER, § p = 0.017 RER vs. DER).

One way ANOVA with repeated measures for GFAP protein levels revealed a statistically significant group effect (F_2,6_ = 21.848, p = 0.002) with the RER group having higher levels of GFAP in both sides of the hippocampus, compared to both the CTR and the DER groups (post hoc, *p = 0.002 CTR vs. RER, § p = 0.001 RER vs. DER).

Similar analysis for DRP1 protein levels showed a statistically significant side × group interaction (F_1,6_ = 74.546, p<0.001). Further post-hoc analysis indicated that in both the control and the RER group DRP1 levels were higher in the right than in the left hippocampus († p<0.05 for both groups) while no laterality was observed in the DER group. In addition, in the right hemisphere DRP1 levels differed between the 3 groups with the highest levels found in the CTR and the lowest in the DER group (post-hoc, *p = 0.011 CTR vs. RER, # p = 0.001 CTR vs. DER, and § p = 0.049 RER vs. DER).

The same statistical analysis for HSP70 protein levels revealed a statistically significant side × group interaction (F_1,6_ = 23.308, p = 0.001). Post-hoc analysis verified in the DER group a significant lateralized expression of HSP70, with higher levels in the right compared to the left hippocampus († p = 0.011). Moreover, in both right and left hippocampi, HSP70 levels were higher in the RER group compared to both the CTR and DER (for the right hemisphere, post hoc, *p<0.001 CTR vs. RER, § p = 0.017 RER vs. DER; for the left hemisphere, *p = 0.033 CTR vs. RER, § p<0.001 RER vs. DER). Also, HSP70 levels differed between control and DER animals in both sides of the hippocampus: In the right side DER animals had higher levels (# p = 0.011) while in the left side the opposite was true (# p = 0.001).

The majority of differences revealed by the proteomic analysis were confirmed by the western blotting. Lack of complete agreement between the results of the proteomic analysis and those of the western blotting could be partially due to the fact that the linearity in the dynamic range of the Coomassie-Blue assay is restricted. Furthermore similar inconsistencies have also been reported by others [8], [30].

## Discussion

The results of the present work clearly demonstrate that the rat hippocampus is lateralized with regard to protein expression levels, as early as 13 days of age and that this laterality can be influenced by early experiences.

The proteins whose expression level was found to differ either between the three experimental groups (DER, RER, CTR) or between the left and right hemisphere belong primarily to three functional classes: cytoskeletal (34%), proteins involved in intermediary, energy metabolism (32%), and in detoxification including chaperones and heat shock proteins (17%), while other categories such as proteins involved in signal transduction (5%), vesicle trafficking (5%) or protein synthesis or degradation (4%), are represented in smaller percentages of the total. Notably similar categories of proteins have been found to differ in their expression level following maternal separation [8], [9], chronic stress [50], [51], [52] and in the schizophrenic human brain [53], [54]. It should be noted that in our study, as in other similar ones using proteomic analysis based on 2-D gels [30], [55], there is a bias towards abundant, soluble proteins, with intermediate molecular weights. Thus, possible differences in proteins of low abundance, or with low or high molecular weight could have been missed, due to the limitations of the analytical method.

Among cytoskeletal proteins, tubulins represent the single protein family whose expression was found to differ most often between the two hemispheres and the experimental groups (17%). Tubulins are the building blocks of microtubules, a major component of the cytoskeleton, which is the cellular structure supporting neuronal axons and dendrites and astrocytic process. It is well known that microtubules undergo growth and reorganization during development and as part of the cellular processes underlying neuronal plasticity. Furthermore, microtubules are the structural components of cilia, which are known to play a role in visceral organ asymmetry, but not in establishing brain laterality at least in humans [31], while in the zebrafish both brain and body asymmetry are affected by altered cilia function [56]. Most interestingly, tubulins were found to have higher levels in the left than the right hemisphere, in all experimental groups, indicating that at this developmental stage there were more growth- and plasticity-related processes going on in the left hemisphere. Relevant to this point is also the finding that tubulins had higher levels in the left hippocampus of the DER animals, compared to that of the RER. Previous studies from our laboratory have indicated that the DER early experience could have analogies to the human situation where the mother is present but unavailable to the child [10]. It is thus interesting to note that tubulin expression has been reported to change in the rat hippocampus following isolation rearing [57], and other stressful experiences, such as chronic unpredictable mild stress [52] or restrain stress [58]. Moreover, changes in the levels of the tubulins have been revealed by proteomic analysis in the hippocampus and cortex, following maternal separation in rats [8], as well as in postmortem tissues from patients with psychiatric diseases [53], [59], indicating the importance of microtubular dynamics in stress-induced pathology and the aitiopathogenesis of psychiatric disorders.

Another cytoskeletal protein whose levels were also found to be higher in the left, compared to the right hippocampus (of the CTR and DER animals), was β-actin, the building block of microfilaments, the supporting structure of dendritic spines, which are reorganized during plasticity-related processes, further supporting that such processes are occurring more in the left hippocampus. Notably actin expression has been shown to change in the rat hippocampus, following maternal separation [8]. Other cytoskeletal proteins functionally involved in neuronal growth, development, regeneration and plasticity were also found to have higher levels in the left compared to the right hippocampus, albeit not in all groups. One of them is α-internexin, an intermediate filament component found in the post-synaptic density, whose levels were higher in the left compared to the right hippocampus of the CTR and RER animals. This finding also supports the view (expressed above) that there are more growth and developmental processes, including new synapse formation, occurring in the left hippocampus, since it has been proposed that synaptogenesis is accompanied by intermediate filament remodeling [60]. Other protein belonging to same class are neurofilament light and medium chains, whose expression also showed a left-right asymmetry in the animals exposed to the early experience (RER and DER). In addition, neurofilament light chain, as well as F-actin capping protein, which regulate actin polymerization and/or depolymerization, also differed between the two experimental groups, showing higher levels in the RER compared to the DER. An additional cytoskeletal protein, worth mentioning is Glial Fibrillary Acidic Protein (GFAP), which was found to be up-regulated in the hippocampus of the RER rat pups. Astrocytic intermediate filaments are composed of GFAP, which is thus a marker of astrocytes [61]. Since astrocytes develop later, our present finding could suggest that the RER early experience might accelerate hippocampal development.

Dihydropyrimidinase like protein (DRP) 1, 2, 3, 4 and 5 comprise another group of proteins, whose expression levels were lateralized, and/or was affected by the early experiences of our model. More specifically DRP 1, 4, and 5 levels were higher in the right compared to the left hippocampus of the RER and CTR rat pups; DRP 3 showed the opposite asymmetrical expression (higher in left than right) in the DER group, while DRP 2 expression was not lateralized. Furthermore, differences in protein levels were detected among the experimental groups (DER, RER, CTR): The levels of all 5 DRPs were lower in the right hippocampus of the DER pups, while in their left hippocampus, DRP 2 and 3 levels were higher than the respective of the RER. DRPs play a role in neurogenesis, neuron differentiation and migration, axon extension and guidance, as well as neuronal morphogenesis. These critical for neuronal development roles might be related to the fact that the levels of these proteins are found to be affected by maternal separation [8], [9], repeated psychological stress [51], anxiety [62], as well as in both the cortex and hippocampus from patients suffering from bipolar or major depression, respectively [53], [59].

Interestingly, in most proteomic analysis studies, such as those investigating the effects of maternal separation [8], [9], chronic stress [51], or mood disorders [59], [63] proteins which play a role in intermediary and energy metabolism appear to be up-, or down-regulated. In our study, in agreement with the above, these include ATP synthase subunits, creatine kinase, dihydrolipoyl dehydrogenase (part of the pyruvate dehydrogenase complex), among others. It appears that the high energy demands of the brain render it very sensitive to experimental manipulations or pathological processes, resulting in changes in the expression of enzymes involved in energy production. Among such enzymes it is worth pointing out glutamate dehydrogenase, which also plays a role in the metabolism of the major excitatory neurotransmitter, glutamate. We found that its levels were higher in the left hippocampus of the DER, compared to those of the CTR, while in the right hippocampus the RER had lower levels than the DER.

In agreement with Marais et al. [9] and Carboni et al. [48] we found differences in the levels of heat shock proteins (HSPs) between the experimental groups. In addition HSPs were found to show left-right asymmetry in their expression. HSPs act as chaperones, either targeting proteins to their cellular destination, or removing abnormal, misfolded or partially degraded proteins. Among the HSPs, Hsp71 cognate protein was found to have higher levels in the left, compared to the right hippocampus, in all experimental groups, indicating more active protein trafficking as part of developmental processes in the left hippocampus, in agreement with our results on tubulins and α-internexin (see above). This protein binds to nascent polypeptides to facilitate correct protein folding. It also functions as an ATPase in the disassembly of clathrin coated vesicles during transport of membrane components through the cell. On the other hand, another member of the HSP70 family, HSP70, which is known to act as an anti-apoptotic chaperone, had higher levels in the hippocampus of the RER pups. Furthermore, in the DER pups, HSP70 showed a lateralized distribution, with higher levels in the right compared to the left hippocampus.

Proteins involved in detoxification of oxygen reactive species, such as superoxide dismutase and protein DJ-1, mutations of which have been identified in patients with amyotrophic lateral sclerosis or Parkinsońs disease, respectively, were found in our study to show differences in their levels between the experimental groups. More specifically, protein DJ-1, whose levels were found to be decreased following maternal separation [9], was up-regulated in the left hippocampus of the pups exposed to either of the two early experiences (DER or RER). Since DJ-1 protein has been proposed to act against dopamine toxicity [64], our finding could indicate that exposure to the early experiences of our model might have a neuroprotective effect on the developing dopaminergic system.

Signal transduction pathways are key players in developmental and plasticity-related processes, and thus the levels of the proteins involved in these pathways, such as 14-3-3, have been found to be altered after maternal separation [8], or chronic stress [51] in the rat hippocampus and cortex, as well as in post mortem brain from schizophrenic patients [53], [65]. In analogy with these studies we found that the levels of 14-3-3 protein were higher in the left hippocampus of the RER compared to that of the DER. Furthermore 14-3-3 had higher levels in the left hippocampus of the CTR pups compared to the right, in support of the view that there were more development- and plasticity-related processes occurring in the left than the right hippocampus (see above). Notably 14-3-3 proteins, in addition to signal transduction, are involved in cell division, differentiation and apoptosis. Furthermore they have been shown to interact with the cytoskeleton in mediating neuronal plasticity [66].

It is worth viewing our results regarding the CTR 13-day-old rat pups in relation to the findings of Samara et al. [30] who investigated, using a proteomics approach, the lateralized protein expression in the adult rat hippocampus. Thirteen of the proteins whose expression differed between the two hippocampi of the control pups also showed lateralized expression in the adult hippocampus. However, in our work the majority of proteins differentially expressed were more abundantly expressed in the left hippocampus, while the opposite was shown by Samara et al. (2011) in the adult rat hippocampus: Many more proteins were highly expressed in the right hippocampus, and fewer in the left [30]. More specifically, the left over right asymmetry in expression found at 13 days of age was maintained into adulthood only for three proteins (HSP 90-α, HSP 90-β and Transitional endoplasmic reticulum ATPase), while only one (Rho GDP-dissociation inhibitor 1) showed a right over left asymmetry in expression in both the neonatal and adult hippocampus. Interestingly, for the majority of proteins (9) asymmetrically expressed both in the 13-day-old and adult hippocampus, the asymmetry in their expression was reversed: For 8 proteins up-regulation was found in the left 13-day-old hippocampus, while in the adult in the right (Table 13), and for only one protein (Peroxiredoxin 2), it was *vice versa*. This shift from left over right to right over left asymmetry in protein expression could reflect developmental changes in laterality, previously shown to occur at the level of gene expression by Moskal et al. [33]. Notably in humans the right cortex develops before the left [67] and a right to left shift in cortical hemispheric asymmetry seems to occur during development: Visuospatial and language abilities, which are localized in the left hemisphere in the adult, have been shown to be localized in the right in infants [68]. An analogous developmental left to right shift could occur in the rat hippocampus, with regard to protein expression, which would account for the difference between our results and those of Samara et al [30]. Such a developmental shift in hippocampal asymmetry would also explain the discrepancy between our results showing a left “dominance” as to the levels of proteins expressed in the 13-day-old hippocampus, while in other studies the long-term effects of early experiences on adult brain laterality show primarily a right hemispheric bias [37], [41], [43], [44].

In conclusion our study shows that protein expression is lateralized in the hippocampus of 13-day-old rat pups and most of the proteins are up-regulated in the left hippocampus. The majority of proteins whose levels differ either between the two hemispheres or between the three experimental groups are cytoskeletal or enzymes involved in energy metabolism. However we also detected differences in the levels of certain proteins that, because of their critical role in neuronal function, seem to be susceptible to changes in expression levels, such as for example the DRPs, the HSPs, or protein 14-3-3. The levels of these proteins have also been shown to be modified following maternal deprivation, another model of early experience.

Interestingly, our work is the first to show that an early experience can alter the degree of hippocampal laterality in protein levels: In the control pups (CTR) the ratio of the number of proteins up-regulated as well as that of the level of protein expression in the left hippocampus to that in the right were 25/7 = 3.6 and 6 respectively. The values of the above described ratios were found for the pups denied the expected reward (DER) to be similar to those of the controls (24/7 = 3.4 for the number of up-regulated proteins and 4.7 for the levels of protein expression). However, for the pups receiving the expected reward (RER) the left to right ratio of the up-regulated proteins was about half (18/10 = 1.8) of that of the controls and the DER, while the ratio of protein expression levels was even lower, approximately less than 1/4 of that of the other two groups (1.1). These results demonstrate that the RER pups exhibited less left to right asymmetry. If during development there is a shift from left to right hippocampal dominance regarding protein expression, as suggested above, the lower degree of laterality in the RER pups could be an indication that the RER experience involving receipt of the expected reward of maternal contact accelerated this developmental shift.
